# A spouse’s perspective on communication breakdowns and supportive strategies for semantic variant primary progressive aphasia

**DOI:** 10.1002/alz.094819

**Published:** 2025-01-09

**Authors:** Surani G Nakkawita, Rene L Utianski

**Affiliations:** ^1^ Mayo Clinic, Rochester, MN USA

## Abstract

**Background:**

The language symptomology associated with semantic variant primary progressive aphasia (svPPA), namely word finding difficulties with loss of word meaning, leads to multimodal communication difficulties. Given that communication plays an intricate role in establishing and sustaining relationships, svPPA can impact relationships, including those with spouses, in a manner that can only be understood by firsthand experiences. This study aimed to describe a spouse’s experiences of communicating with an individual with svPPA along with the role of communication supports and expectations for speech‐language therapy.

**Method:**

A semi‐structured interview using open‐ended questions was conducted with the spouse of an individual with svPPA. The interview transcript was reviewed and analyzed using thematic analysis by both authors.

**Result:**

The themes that emerged when discussing living with an individual with svPPA included: the role of communication in their prior life; changes in communication and behavior; emotional impact; adjustments and accommodations; and community engagement and supports. Themes associated with communication supports included: external tools; adaptive strategies; individualization and adaptation; and utility. The spouse in the current study described a tool, specifically a notebook in the form of a dictionary that was initiated and developed by the individual with svPPA. The tool increased his confidence in communication as it aided comprehension and recognition of words. Caregiver inclusion, learning about the patient, and evolving strategies are themes surrounding the spouse’s expectations from speech therapy.

**Conclusion:**

The study revealed challenges in the spouse of an individual with svPPA understanding and expressing thoughts to her partner, which resulted in feeling more distant as well as changes in social dynamics. The study also describes the benefits of communication supports for both the spouse and the individual with svPPA. Additionally, the study revealed the important role the SLP, and the community plays in enhancing well‐being. Although the findings from the current study cannot be ubiquitously generalized, it provides insight into the changing dynamics of a couple navigating svPPA and reiterates the importance of including patients and their care partners in treatment planning and the benefit of communication supports and speech therapy across the disease course.

